# Foodborne Pathogen Biofilms: Development, Detection, Control, and Antimicrobial Resistance

**DOI:** 10.3390/pathogens12020352

**Published:** 2023-02-20

**Authors:** Kidon Sung, Saeed Khan, Juhee Ahn

**Affiliations:** 1 Division of Microbiology, National Center for Toxicological Research, Food and Drug Administration, Jefferson, AR 72223, USA; 2 Department of Medical Biomaterials Engineering, Kangwon National University, Chuncheon 24341, Gangwon, Republic of Korea

Bacteria can grow either as planktonic cells or as communities within biofilms. The biofilm growth mode is the dominant lifestyle of most bacterial species and 40–80% of microorganisms are associated with biofilms [1]. Biofilm is a sessile community that is irreversibly attached to a substratum or interface or to other members of the community [2]. It is surrounded by extracellular polymeric substances (EPS) that include extracellular polysaccharides, extracellular DNA, lipids, proteins, and other elements [3]. Biofilm formation is a complex but well-regulated process that can be classified into five distinct stages [4]. In the first stage, planktonic bacteria attach to a surface. *Salmonella* species, *Listeria monocytogenes*, *Campylobacter jejuni,* or *Escherichia coli* have specific structures on the surface of the bacteria, such as flagella, curli, fimbriae, and pili, which help the bacteria attach [5]. The second stage is the adhesion step, which includes an initial reversible adhesion resulting in loose adhesion and a subsequent irreversible adhesion resulting in more stable adhesion. The third stage is to secrete EPS and form microcolonies. This is followed by biofilm maturation, which produces large amounts of EPS to grow in size and build three-dimensional structures. The final stage is the stage in which the biofilm is dispersed, releasing the planktonic cells and initiating the formation of a new biofilm at another location.

Microbial cells living within biofilms are protected from various environmental stresses such as desiccation, osmotic changes, oxidative stress, metal toxicity, radiation, antibiotics, disinfectants, and the host immune system [6]. Biofilms are much less sensitive to antimicrobial agents than planktonic cells, and several mechanisms contribute to their resistance to antimicrobials [7]. The exopolysaccharide matrix prevents the entry of antimicrobial agents by reducing diffusion and acting as a primary barrier [8]. Most antimicrobial agents kill rapidly dividing cells more effectively, but slow growth of biofilms leads to resistance [9]. Changes in metabolic activity within biofilms, genetic changes of antimicrobial resistant determinants in target cells, extrusion of antimicrobial agents using efflux pumps, and the presence of persistent cells also contribute to antimicrobial resistance [10].

Foodborne pathogens, such as *L. monocytogenes*, *Salmonella* spp., *E. coli* O157:H7, *C. jejuni*, *Clostridium perfringens*, *Bacillus cereus*, and *Staphylococcus aureus*, and food spoilage bacteria, such as *Pseudomonas* spp., *Lactobacillus* spp., and *Shewanella* spp., can produce biofilms and are an important food safety issue causing huge economic losses in the food industry [11,12]. The extracellular matrix of biofilms can adhere to hard surfaces on food processing equipment or to food contact surfaces and serves as a structure in sustaining these biofilms [13]. Biofilms protect spoilage and foodborne pathogens in the cleaning processes in food processing equipment, such as drying or treatment with disinfecting agents [11]. Biofilms of spoilage and foodborne pathogens that survive in the sanitizing step may contaminate food products, shortening shelf life and causing food poisoning [12]. The risk is compounded by the fact that cells in biofilms have been shown to have increased resistance to sanitizing agents compared to planktonic cells [14]. *L. monocytogenes* induce biofilm formation in response to low temperatures, which increase adhesion to surfaces and resistance to disinfecting treatment in many food manufacturing plants [15]. *L. monocytogenes* biofilms exposed to commercial disinfectants, including quaternary ammonium compounds, have increased resistance to disinfectants [16]. In addition, benzalkonium chloride-adapted *S*. Enteritidis biofilms can develop resistance more efficiently than planktonic counterparts [17]. Ju et al. reported an approximately 32,768-fold increase in ampicillin resistance in *S*. Dublin biofilms compared to planktonic cells [18]. González et al. found that *S*. Typhimurium biofilms were significantly more resistant to ciprofloxacin both in vitro and in vivo [19].

This Special Issue aims to discuss recent studies on biofilm formation/development, detection techniques, prevention and control measures, and antimicrobial resistance associated with foodborne pathogens. This Special Issue comprises five original research articles with contributions by 26 authors from China, Iraq, Italy, Korea, Poland, Russia, South Africa, and the USA, and two review articles by four authors from the USA.

By forming biofilms, *L. monocytogenes* can survive for long periods in food processing plants, contaminating food at various stages of production [20]. The two research articles highlight the variability in the biofilm production among *L. monocytogenes* strains collected from different sources such as food and food production environments and the prevalence of biofilm-associated genes. Wiśniewski et al. investigated biofilm formation potential and the prevalence of biofilm-forming genes (*inlB, luxS, sigB*) among *L. monocytogenes* isolated from food and food processing environments in Poland [21]. Strains isolated from food processing environments formed biofilms at a higher frequency than strains isolated from food, and *inlB, luxS*, and *sigB* were detected in all strains from food processing environments. Kaptchouang Tchatchouang et al. also evaluated the biofilm formation ability and the frequency of biofilm-associated genes (*flaA, luxS*) in *L. moncytogenes* isolated from South African food samples [22]. They found that all isolates consistently and strongly formed biofilms at 4 °C for 24, 48, and 72 h. Biofilm-forming genes *flaA* and *luxS* were detected in 72% and 56% of the isolates, respectively.

It is urgent to take effective novel strategies in preventing and eradicating biofilms to reduce the risk of microbial infection. Combining anti-biofilm agents, such as quorum sensing inhibitors, probiotics, bacteriophages, and antimicrobial peptides, with antibiotics is emerging as a promising strategy to eradicate biofilms [23]. Three research articles in this Special Issue investigated a combination treatment of antibiotics with β-lactamase inhibitors, efflux pump inhibitors, and probiotic bacteria against bacterial biofilms. Laure and Ahn studied the anti-biofilm effect of β-lactam and β-lactamase inhibitor combinations against antibiotic-sensitive and multidrug-resistant *S.* Typhimurium [24]. They discovered that a combination of a β-lactam (ampicillin, ceftriaxone) and a β-lactamase inhibitor (sulbactam) significantly inhibited biofilms of β-lactamase-producing multidrug-resistant *S.* Typhimurium. Dawan et el. assessed the effect of an efflux pump inhibitor on *S.* Typhimurium biofilm formation [25]. They discovered that combinations of antibiotics (ceftriaxone, chloramphenicol, ciprofloxacin, erythromycin, norfloxacin, tetracycline) and an efflux pump inhibitor (phenylalanine-arginine β-naphthylamide) synergistically suppressed quorum sensing and thus *S.* Typhimurium biofilm formation. AL-Dulaimi et el. investigated anti-biofilm activity of a mixture of a cationic antimicrobial peptide (polymyxin E) and probiotic bacteria, such as *Bacillus subtilis* KATMIRA1933 and *Bacillus amyloliquefaciens* B-1895, against *Acinetobacter* species [26]. Their results exhibited a significant inhibition of biofilm formation in *Acinetobacter* species when the cell-free supernatants of *Bacillus* strains were combined with polymyxin E, compared to the use of the antibiotic alone.

Although conventional approaches are being employed to kill the biofilms of foodborne pathogens, they are still ineffective, and more new innovative agents capable of controlling biofilms are required [27]. In particular, in the food industry, there is demand for natural compounds that can be safely added to food products to act as a biofilm remover, as well as curtailing spoilage and preventing food contamination. Esposito and Turku bring a comprehensive review on innovative natural methods of targeting foodborne pathogens’ biofilms, including bacteriocins, bacteriophages, fungi, phytochemicals, plant extracts, essential oils, gaseous and aqueous control, photocatalysis, enzymatic treatments, and ultrasound [28]. Understanding how foodborne pathogens form and survive in food processing environments is important for developing new strategies against sanitizer resistance and repeated contamination. Studies related to biofilm control of foodborne pathogens are primarily designed for single-species biofilms and ignore the fact that biofilms exist as mixed-species biofilms in most food processing environments. Dass and Wang draw attention to the potential food safety issues associated with disinfecting agents that control mixed-species biofilms in the food processing environments [29].

We are grateful to all the authors that provided valuable research findings and updated reviews. We hope that this Special Issue will be an essential resource for understanding the biofilms of foodborne pathogens and developing their mitigation strategies.
